# The Effect of Time Pressure on Risky Financial Decisions from Description and Decisions from Experience

**DOI:** 10.1371/journal.pone.0123740

**Published:** 2015-04-17

**Authors:** Pete Wegier, Julia Spaniol

**Affiliations:** Department of Psychology, Ryerson University, Toronto, Ontario, Canada; US Army Engineer Research and Development Center, UNITED STATES

## Abstract

Time pressure has been found to impact decision making in various ways, but studies on the effects time pressure in risky financial gambles have been largely limited to description-based decision tasks and to the gain domain. We present two experiments that investigated the effect of time pressure on decisions from description and decisions from experience, across both gain and loss domains. In description-based choice, time pressure decreased risk seeking for losses, whereas for gains there was a trend in the opposite direction. In experience-based choice, no impact of time pressure was observed on risk-taking, suggesting that time constraints may not alter attitudes towards risk when outcomes are learned through experience.

## Introduction

Often judgments and decisions are made without the benefit of unlimited periods of time for deliberation [1]. We may be allowed to ponder our options for some time or have to make decisions in the blink of an eye. Time pressure is experienced when the time to make a judgment or decision is constrained. As time pressure increases decisions may change—because there is less time to learn about choice options, because different aspects of the choice become more salient, or because different cognitive strategies are employed. This is especially important in areas such as financial decision-making—the buying or selling of goods or trading on the stock market—where outcomes may be uncertain and opportunities may be time-sensitive. One topic that remains understudied is how time pressure impacts decision-making regarding monetary outcomes, specifically in choices where outcomes are risky and cannot be predicted in advance.

Pressure—or stress in general—impacts cognitive performance in various ways. Early studies demonstrated that time pressure can bias judgments toward already known information and away from externally provided information, and that it can influence the weighting of positive and negative information [2,3]. Stress has also been shown to modulate learning about choice outcomes, improving learning if the outcomes were positive, but impairing learning when outcomes were negative [4]. In one study, time pressure increased reliance on negative information in a consumer choice task. Participants were provided with information about various cars and were required to choose a car under various levels of time pressure [5]. A data-usage model revealed that as time pressure increased, participants put greater weight on the negative information provided and less on the positive information when making their decisions. Similar emphasis on negative information has been observed in a choice task involving risky financial gambles [6]. Participants were given a series of binary choice problems under three levels of time pressure and it was observed that increased time pressure resulted in a decrease in risk-taking, with participants favouring the option with the lower variance (or smaller amount to lose). The authors speculated that reduced risk-taking diminishes the stress associated with time pressure. However, others have suggested that the observed decrease in risk-taking under time pressure depended on the expected values of the outcomes [7,8]. In two studies which presented participants with choices between certain and uncertain payoffs, risk-taking increased under time pressure when expected values were positive, but decreased if expected values were negative. Finally, the pressure created by one’s own expectations can modulate outcome learning. For example, Hommes and colleagues have demonstrated that individuals in economic pricing experiments extrapolate expected price increases and decreases into the future, resulting in pricing bubbles [9,10].

Past research has shown time pressure can affect strategies used during decision making [11,12], with different intensities of time pressure resulting in varied adaptions to those constraints and subsequently differing approaches to judgments and decisions [2]. Additionally, there is evidence that the imposition of a time constraint may prevent individuals from making advantageous decisions by reducing cognitive flexibility [13]. In one study, participants were given two rating tasks in which they had to rate either the attractiveness or buying price of risky monetary gambles, with each requiring different cognitive strategies. Some individuals failed to switch strategies under time pressure, suggesting that the presence of a time constraint may have diminished cognitive flexibility. In a test of mathematical problem-solving it was found that a combination of social pressure, monetary rewards, and implicit time constraints led to performance decrements, at least in those with high working-memory capacity [14]. More recently, time pressure has been shown to impair performance on experience-based decision-making tasks, such as the Iowa Gambling Task [15]. In more ecologically valid situations—such as behaviour during a fire—it has been shown that time pressure and stress reduce the utilization of environmental cues, spatial awareness, and the likelihood of escaping the burning area [16].

Sometimes choice outcomes and risks are explicitly provided to us—known as *decisions from description*—whereas other times we must rely on information learned by experiencing the outcomes of those choices—known as *decisions from experience*. The literature has found that individuals tend to overweight the impact of rare events in description-based choice but underweight their impact in experience-based choice [17], leading to a reversal of preferences termed the *description-experience gap*. A variety of mechanisms, such as recency effects and sampling error in experience-based choice [18] have been implicated in the description-experience gap. Hau et al. [19] found that memory—manipulated via pre-determined sample sizes—had little effect on performance and that observed differences in choice were largely the result of format of choice presentation. What is currently unknown is how task constraints such as time pressure affect description- and experience-based choice, respectively. As making decisions from description and experience differs in regards to cognitive mechanisms involved, such as memory for outcome learning, the effect of time pressure may differ between the two choice formats.

In summary, time pressure appears to have an influence on risky choice, but results have been somewhat mixed [6,20]. Additionally, time pressure has been shown to increase focus on negative information prior to a choice [5]. These findings, combined with the lack of studies examining time pressure in experience-based choice, provided the motivation for the current work. To increase the generality of time pressure findings we examined both decisions from description and decisions from experience. Participants completed a computerized risky financial choice task, consisting of choices between smaller-but-certain and larger-but-risky monetary rewards, in high and low time-pressure conditions. In Experiment 1, choice options were presented in a description format and time pressure was manipulated within subjects. In Experiment 2, a sampling procedure was used to present choice options in an experience format and time pressure was manipulated between subjects. Our study was a partial replication of Hau et al. [19]—with the addition of a time pressure manipulation and loss trials in the choice problem set. We elected to use these choice problems as they were designed to manipulate outcome probabilities factorially, for both very low and very high probabilities where differences between description- and experience-based formats have previously been observed [19]. Participants additionally completed a battery of self-report measures to investigate any characteristics that may correlate with risk-taking or information search. Together, these experiments addressed the following research questions:

### Framing

Does time pressure affect risk-taking and does this effect depend on the framing of the options (i.e., gain vs. loss)? Based on past findings [7,8] we hypothesized that time pressure would increase the proportion of risky choices for monetary losses but would have a smaller or no effect on risky choices for monetary gains.

### Adaptivity

Do participants modulate risk-taking or information search according to the probability of the desirable outcome? Adaptivity cannot be assumed—for example, there is evidence of age-related impairments to feedback learning [21], which is necessary in experience-based choice. Similar observations of *adaptive decision-making* [22] and *adaptive sampling* [23] have been made in past studies. Therefore we predicted that participants would engage in greater risk-taking and decreased information search as the probability of the desirable outcome increased. As past studies found that participants spent more time looking at negative information [5,6,20] we hypothesized that participants would sample more on loss trials (undesirable or negative outcomes) than on gain trials (desirable or positive outcomes) in decisions from experience, particularly under time pressure. We did not have a specific hypothesis in regards to the interaction of time pressure and the probability of the desirable outcome.

## Experiment 1

### Method

#### Participants

Participants included 40 undergraduate students (7 male) who received course credit in exchange for their participation. Participant characteristics are shown in Table 1. An additional nine participants were tested but excluded from analysis due to self-reported history of major health problems that may interfere with cognitive function (e.g., head injury, history of psychiatric illness). This research was approved by the Research Ethics Board at Ryerson University. In accordance with research ethics guidelines at universities in Canada, all undergraduate student participants over the age of 16 are considered ‘adults’ with the capacity to consent to research [24]. This consent procedure was also reviewed and approved by the Research Ethics Board at Ryerson University. All participants provided written informed consent to participate in the study.

**Table 1:** Participant characteristics.

Characteristic	Experiment 1 (*N* = 40)	Experiment 2: High Time Pressure (*N* = 24)	Experiment 2: Low Time Pressure (*N* = 25)
Age (years)	20.1 (4.7)	21.1 (3.0)	20.6 (3.0)
Age range	17–41	18–30	18–30
Education (years)	13.5 (1.2)	14.4 (1.4)	14.2 (1.2)
Self-Control*	117.8 (14.2)	122.7 (12.1)	131.8 (12.1)
Risk Propensity	28.4 (6.6)	30.0 (6.6)	26.4 (9.1)
Numeracy	8.4 (1.8)	8.3 (2.0)	9.2 (1.7)
Need for Cognition	9.2 (4.7)	9.9 (3.9)	9.2 (4.7)
BIS	–	22.2 (3.4)	21.4 (3.1)
BAS Drive	–	11.6 (2.6)	11.5 (2.0)
BAS Fun Seeking	–	12.00 (1.7)	11.6 (2.4)
BAS Reward Responsiveness	–	17.5 (1.8)	17.8 (1.8)

*Note*. *Difference between participant groups in Experiment 2, *p* < .05

#### Design

Participants completed a risky financial choice task that required them to choose between smaller-but-certain and larger-but-risky monetary rewards (e.g., $1.00 for sure or a 20% chance of $4.00, else $0). The design included four within-subjects factors: (1) time pressure (high or low); (2) framing (gain or loss); (3) the probability of the desirable outcome (*p*_desirable_: 0.10, 0.20, 0.80, 0.90); and (4) the payoff variability of the risky option ($1.60, $4.50, $9.60). The proportion of risky choices made served as the dependent variable.

Pilot testing without time constraint showed that response times (RTs) were highly variable between participants. Therefore, time constraints for the time pressure condition were individually determined on the basis of a 10-trial practice run. Each participant’s 75^th^ percentile RT from the practice task was then used as the time limit in the time pressure condition. The order of the time pressure conditions (high vs. low) was counterbalanced across participants.

#### Stimuli

The risky financial choice task included the 12 choice problems from Hau et al. [19] Experiment 1, shown in Table 2. Each problem required participants to choose between a risky option (*X* with probability *p*_non-zero_ or $0 with probability 1 − *p*_non-zero_) and a certain option (*Y* with probability 1.0). The value of *Y* was either slightly above or below the expected value of the risky option. Half of the problems used the smaller value of *Y* while the other half used the larger value. The assignment of each was counterbalanced across participants. The original protocol used only choices involving gains but we presented participants with both gain and loss choices [23]. The loss choices were identical to the gain choices but involved negative outcomes (e.g., −$1.00 for sure or a 20% chance of −$4.00, else $0). All data analyses were carried out with the probability of the non-zero outcome (*p*_non-zero_) recoded as *p*_desirable_ (the probability of the desirable outcome). In order to maximize desirable outcomes, one should choose the risky option when *p*_non-zero_ is high during gain trials, whereas one should choose the risky option when *p*_non-zero_ is low during loss trials. Therefore, *p*_desirable_ allows for more meaningful comparisons than *p*_non-zero_. In gain trials, *p*_desirable_ = *p*_non-zero_, whereas in loss trials, *p*_desirable_ = 1 − *p*_non-zero_.

**Table 2:** Choice problems.

Choice	Risky option: *X* (in CAD)	Risky option: *p*_non-zero_	Risky option: Variability	Certain option: *Y* (in CAD)
1	$5.30	0.10	$1.60	$0.30 / $0.70
2	$4.00	0.20	$1.60	$0.60 / $1.00
3	$4.00	0.80	$1.60	$3.00 / $3.40
4	$5.30	0.90	$1.60	$4.60 / $5.00
5	$15.00	0.10	$4.50	$1.30 / $1.70
6	$11.30	0.20	$4.50	$2.10 / $2.50
7	$11.30	0.80	$4.50	$8.80 / $9.20
8	$15.00	0.90	$4.50	$13.30 / $13.70
9	$32.00	0.10	$9.60	$3.00 / $3.40
10	$24.00	0.20	$9.60	$11.80 / $12.20
11	$24.00	0.80	$9.60	$19.00 / $19.40
12	$32.00	0.90	$9.60	$28.60 / $29.00

*Note*. Each problem required participants to choose between a risky option (*X* with probability *p*_non-zero_ or $0 with probability 1 − *p*_non-zero_) and a certain option (*Y* with probability 1.0). The value of *Y* was either slightly above or below the expected value of the risky option.

To present the stimuli and collect responses, we used MATLAB version R2011B (The MathWorks Inc.), with the Psychophysics Toolbox extension [25] version 3.0.9, running on an Intel Core 2 Quad 2.40 GHz 32-bit Windows Vista desktop computer with 4GB of RAM and a 23-inch LCD display. All text appeared in black a 53-point typeface on a white background.

#### Procedure

Participants were tested individually in a quiet, well lit testing room. After signing a consent form, participants were given instructions for the risky financial choice task and informed that they could receive a monetary reward if their cumulative earnings were greater than $0. Before proceeding, participants completed a practice session consisting of 10 choice problems for which they received no money. Following the practice, participants completed the 24 choice problems, presented in random order, twice: once in a high time pressure block and once in a low time pressure block. The order of the blocks was counterbalanced and participants were informed which they were completing prior to starting each block. For the high time pressure block, participants were each told how much time they had for each choice, based on their practice sessions, and this information was verbally provided to participants several times before the start of the experimental portion of the study. No onscreen countdown was provided during each trial. If they participant took too long to answer the words “NO RESPONSE DETECTED” were displayed on the screen and the program proceeded to the next trial. Choice options were presented side-by-side on the screen and participants responded by pressing whichever response key corresponded to the side on which the option they preferred was displayed. The left/right assignment of each option was counterbalanced across participants. After a choice was made, a fixation-cross appeared for 1,000 ms, followed by the next choice problem. No end-of-trial feedback was given to participants regarding the outcome of their decision or their current total in order to eliminate possible confounds due to wealth effects or affective responses to the outcomes. Once participants had completed all 24 choice problems their cumulative earnings were displayed on the screen. If participants had earnings greater than $0 they were paid out this amount; otherwise, they received (or owed) nothing.

After the choice task, participants completed a series of paper-and-pencil questionnaires, including: the Risk Propensity scale [26], a 7-item questionnaire measuring general, everyday risk-taking; the Numeracy scale [27], an 11-item questionnaire measuring mathematical proficiency pertaining to concepts such as fractions, decimals, percentages, and proportions; the Need for Cognition scale [28], an 18-item questionnaire measuring enjoyment of effortful cognition; and the Self-Control Scale [29], a 36-item questionnaire measuring self-regulatory ability.

### Results

In Experiment 1, we focused on risk-taking as a function of time pressure and choice framing. To examine how these factors interacted with the probabilistic structure of the choice problems, we also analyzed the effects of *p*_desirable_ and payoff variability on risk-taking. Due to the small number of choice problems, separate analyses were carried out in which we collapsed across levels of either *p*_desirable_ or payoff variability. To check the effectiveness of the time pressure manipulation we also analyzed response times (RTs).

#### RT

A 2 (time pressure: high, low) × 2 (framing: gains, losses) repeated measures ANOVA on RT (Table 3) yielded a significant main effect of time pressure, *F*(1, 39) = 45.66, *p* < .001, partial η^2^ = .54, with participants responding faster in the high time-pressure condition (*M* = 2,679 ms) than in the low time-pressure condition (*M* = 4,598 ms). The main effect of framing was also significant, *F*(1, 39) = 13.36, *p* = .001, partial η^2^ = .26, with participants responding faster during gain trials (*M* = 3,237 ms) than during loss trials (*M* = 4,040 ms). This effect was qualified by a significant Time Pressure × Framing interaction, *F*(1, 39) = 11.52, *p* = .002, partial η^2^ = .23. Pairwise comparisons revealed no effect of choice framing on response times in the high time-pressure condition, but a significant effect in the low time-pressure condition.

**Table 3:** Response times (ms).

Condition	Gains	Losses
**Experiment 1**		
High time pressure	2,600 (1,517)	2,758 (1,540)
Low time pressure	3,874 (2,186)	5,321 (3,106)
**Experiment 2**		
High time pressure	9,721 (4,462)	10,080 (4,391)
Low time pressure	16,218 (6,533)	17,481 (7,079)

#### Risk-taking

**Fig 1:**
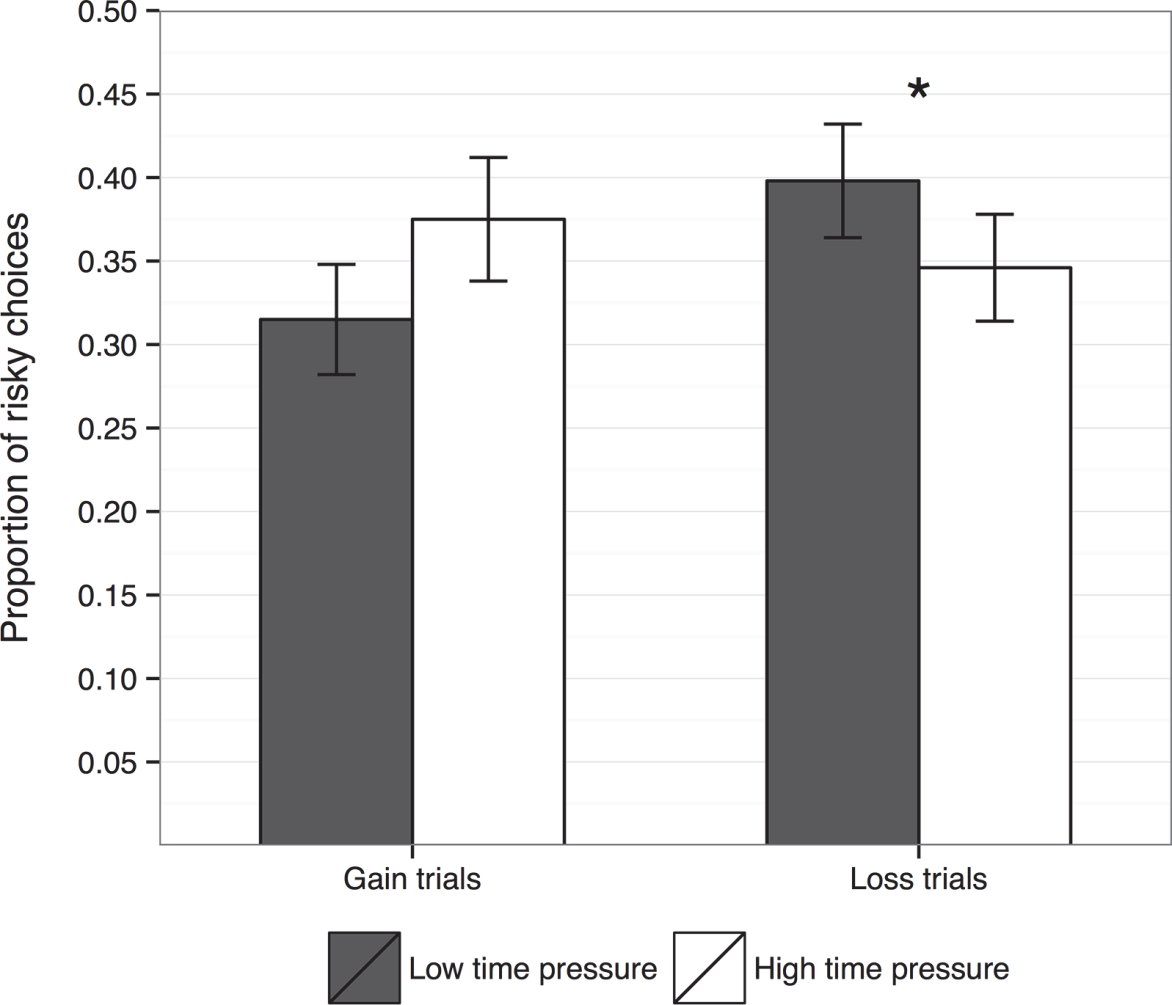
Experiment 1—Proportion of risky choices taken as a function of the choice framing (gains vs. losses) and the time-pressure condition. Error bars indicate standard errors of the mean.

In the first analysis, we collapsed across the levels of payoff variability and conducted a 2 (time pressure) × 2 (framing) × 4 (*p*_desirable_: 0.10, 0.20, 0.80, 0.90) repeated measures ANOVA on the proportion of risky choices. The main effects of time pressure and framing were not significant. However, there was a significant Time Pressure × Framing interaction, *F*(1, 39) = 8.29, *p* = .006, partial η^2^ = .18 (Fig 1). Follow-up *t* tests showed a marginally nonsignificant effect of time pressure on the proportion of risky choices taken for gain trials, *t*(39) = 1.74, *p* = .09, *d* = .25, such that participants showed a tendency to choose the risky option more often in the high time-pressure condition (*M* = .40) than in the low time-pressure condition (*M* = .34). The opposite pattern was observed for loss trials, *t*(39) = 2.13, *p* = .039, *d* = .34, where participants chose the risky option more often in the low time-pressure condition (*M* = .38) than in the high time-pressure condition (*M* = .32). A significant main effect of *p*_desirable_ was found, *F*(1.40, 54.47) = 4.06, *p* = .036, partial η^2^ = .09 (Table 4), after correction for non-sphericity using the Greenhouse-Geisser estimate (ε = .47). This main effect was qualified by two significant interactions. First, the Time Pressure × *p*_desirable_ interaction was significant, *F*(3, 117) = 3.45, *p* = .019, partial η^2^ = .08. However, pairwise contrasts between time pressure conditions for each level of *p*_desirable_ were not significant. Second, the Framing × *p*_desirable_ interaction was significant, *F*(1.96, 76.45) = 6.32, *p* = .001, partial η^2^ = .14, after correction for non-sphericity using the Greenhouse-Geisser estimate (ε = .65). Pairwise contrasts between time pressure conditions for each level of *p*_desirable_ revealed significant differences two levels. At the. 20 level participants took the risky option more often for losses (*M* = .33) than for gains (*M* = .23), while at the. 90 level participants took the risky option more often for gains (*M* = .52) than for losses (*M* = .34).

**Table 4:** Experiment 1—Proportion of risky choices taken for differing levels of *p*_desirable_ and payoff variability.

Level	Condition: High time pressure	Condition: Low time pressure	Framing: Gains	Framing: Losses
***p***_**desirable**_				
0.10	.30 (.26)	.34 (.27)	.33 (.30)	.31 (.30)
0.20	.25 (.23)	.31 (.24)	.23 (.24)	.33 (.27)
0.80	.44 (.28)	.35 (.26)	.40 (.32)	.39 (.30)
0.90	.43 (.28)	.43 (.31)	.52 (.37)	.24 (.31)
**Payoff Variability**				
$1.60	.36 (.18)	.33 (.20)	.38 (.22)	.31 (.22)
$4.50	.34 (.19)	.38 (.22)	.40 (.21)	.32 (.22)
$9.60	.36 (.20)	.38 (.22)	.33 (.21)	.41 (.27)

In the second analysis, we collapsed across the levels of *p*_desirable_ to conduct a 2 (time pressure) × 2 (framing) × 3 (payoff variability: $1.60, $4.50, $9.60) repeated measures ANOVA on the proportion of risky choices. As described in the previous analysis, the main effects of time pressure and framing were not significant but there was a significant Time Pressure × Framing interaction. Regarding the factor of payoff variability, only the interaction of Framing × Payoff Variability was significant, *F*(2, 78) = 9.81, *p* < .001, partial η^2^ = .20 (see Table 4). Pairwise contrasts revealed that the difference in risk-taking between gains and losses at the intermediate ($4.50) payoff variability level was marginally nonsignificant, *t*(39) = 1.98, *p* = .054, *d* = .31. There were no framing effects at low ($1.60) and high ($9.60) levels of payoff variability.

#### Correlations with self-report measures

We examined bivariate correlations of participants’ self-report measures with their risk-seeking behaviour and response times. The only significant finding was the correlation between Need for Cognition (NFC) and overall risk-taking, *r* = .34, *p* = .034. Participants with higher NFC scores displayed greater risk-seeking behaviour.

### Discussion

In this experiment, participants were presented with risky financial choices in both high and low time-pressure conditions. The time-pressure manipulation was successful at modulating behaviour, as indicated by the RT analysis. The effect of time pressure on risk-taking depended on the framing of the choice options. When faced with a potential loss, participants made fewer risky choices under high time pressure than under low time pressure. In contrast, gain trials showed a trend in the opposite direction (more risky choices under low time pressure than under high time pressure). Furthermore, in line with the adaptive decision-making behaviour predicted by our Adaptivity hypothesis, participants chose the risky option more often as the probability of the desirable outcome increased.

As Experiment 1 presented participants with explicit probability and outcome information, we were not able to investigate the effect of time pressure on information search. In order to test whether time pressure changes the degree of pre-decisional information search, we conducted a second experiment in which participants had to learn the outcomes and their probabilities through experience, specifically through a sequential sampling paradigm. Studies comparing description- and experience-based choice have found differences in risky choice behaviour [18]; however, little work has been done to investigate the effects of time pressure on experience-based choice. This is a problem of particular interest as everyday decisions do not often provide *a priori* knowledge of their risks and associated payoffs, and are usually constrained temporally. Thus we sought to investigate the effect of time pressure on experience-based choice.

## Experiment 2

The design of Experiment 2 was identical to that of Experiment 1 with the following exceptions. First, time pressure was manipulated between-subjects instead of within-subjects. Second, the choice problems in the risky financial choice task were presented in an experience-based choice paradigm. Unlike in the description-based paradigm of Experiment 1, no explicit probability information was provided in Experiment 2; however, participants were allowed to learn the probabilities by sampling the risky options.

### Method

#### Participants

Participants included 24 undergraduate students (6 male) in the high time-pressure condition and 25 undergraduate students (8 male) in the low time-pressure condition. Participant characteristics are shown in Table 1. Eleven additional participants were recruited but excluded on the basis of a history of self-reported health problems. All participants gave written informed consent to participate in the study. The study and the consent procedure were reviewed and approved by the Research Ethics Board at Ryerson University.

#### Design

The dependent variables included the proportion of risky choices taken and the sampling frequency as a measure of information search.

#### Procedure

**Fig 2:**
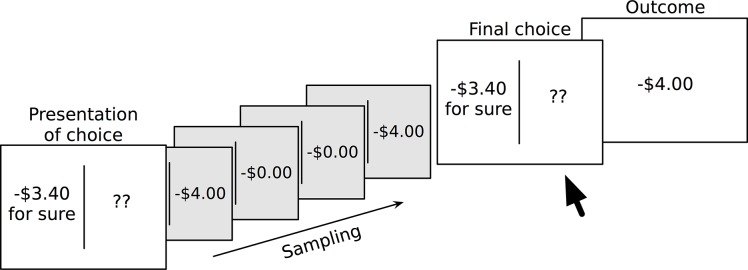
Illustration of the sampling procedure in experience-based risky financial choice task. Here the participant samples a total of four times, seeing −$4.00 and $0 twice each. From this they may conclude there is a 50% chance of losing $4.00 and a 50% chance of losing nothing. The participant decided to choose the risky option and is shown the result, a loss of $4.00.

Fig 2 presents an overview of the modified risky financial choice task. Participants were explicitly provided probability information for the certain option (e.g., “−$3.40 for sure”) but the risky option initially appeared as a pair of question marks. Participants learned outcomes and their probabilities by repeatedly sampling from the distributions of outcomes via a button press. Participants were allowed to sample as many times as they liked—no limit was placed on the number of samples drawn—but they operated under a time constraint if assigned to the high time-pressure condition. Outcomes from sampling were purely informative and had no influence on the participant’s current total. Once the participant had sampled to their satisfaction they pressed a key to indicate they wished to make their final choice and pressed one of two keys to indicate which of the two options (certain or risky) they would prefer. If the risky option was chosen, the outcome would be determined from a random draw of the probability distribution for that choice problem. For the high time pressure condition, participants were each told how much time they had for each choice, based on their practice sessions, and this information was verbally provided to participants several times before the start of the experimental portion of the study. No onscreen countdown was provided during each trial. If they participant took too long to answer, the words “NO RESPONSE DETECTED” were displayed on the screen and the program would move on to the next trial. Once all choice problems were complete the participant’s total was displayed onscreen. Finally, participants completed the same series of paper-and-pencil questionnaires as in Experiment 1, with the addition of the Behavioral Inhibition System (BIS) and Behavioral Activation System (BAS) scales [30]: a 24-item questionnaire measuring behavioural inhibition, fun/novelty seeking, responsiveness to rewards, and motivational drive. The participant groups were matched on all measures except the self-control measure, where those in the low time pressure group (*M* = 131.76) had higher self-reported self-control than those in the high time pressure group (*M* = 122.67).

### Results

#### RT

We performed a 2 (time pressure) × 2 (framing) mixed ANOVA as a manipulation check to examine whether time pressure had an effect on response times (Table 3). We found a significant main effect of time pressure, *F*(1, 47) = 18.69, *p* < .001, partial η^2^ = .28, with participants responding faster in the high time-pressure condition (*M* = 9,726 ms) than in the low time-pressure condition (*M* = 16,850 ms). The main effect of framing was significant, *F*(1, 47) = 4.93, *p* = .031, partial η^2^ = .10, with participants responding faster during gain trials (*M* = 12,970 ms) than during loss trials (*M* = 13,781 ms). The Time Pressure × Framing interaction was not significant.

#### Risk-taking

As Self-Control scores significantly differed between groups we included them as a covariate in each of the analyses described next. In the first analysis, we collapsed across the levels of payoff variability and conducted a 2 (time pressure) × 2 (framing) × 4 (*p*_desirable_) mixed-design analysis of covariance (ANCOVA) with Self-Control as a covariate to investigate risk-taking as operationalized by the proportion of risky choices. The main effects of time pressure and framing were not significant, as in Experiment 1. The Time Pressure × Framing interaction was also not significant. A significant main effect of *p*_desirable_ was found, *F*(1.95, 89.48) = 79.41, *p* < .001, partial η^2^ = .63, after correction for non-sphericity using the Greenhouse-Geisser estimate (ε = .65). This effect was qualified by a significant Time Pressure × *p*_desirable_ interaction, *F*(3, 138) = 2.88, *p* = .039, partial η^2^ = .06 (Table 5). The interaction was driven by the difference between time-pressure conditions at the. 90 level only, *t*(47) = 2.14, *p* = .04, *d* = .61, with participants selecting the risky option more often under high time pressure (*M* = .72) than under low time pressure (*M* = .56). All other interactions were not significant.

**Table 5:** Experiment 2—Proportion of risky choices taken for differing levels of *p*_desirable_ and payoff variability.

Level	Condition: High time pressure	Condition: Low time pressure	Framing: Gains	Framing: Losses
***p***_**desirable**_				
0.10	.18 (.16)	.19 (.17)	.21 (.24)	.16 (.25)
0.20	.16 (.14)	.22 (.20)	.23 (.26)	.15 (.24)
0.80	.65 (.26)	.59 (.27)	.67 (.38)	.58 (.34)
0.90	.72 (.24)	.56 (.26)	.63 (.39)	.64 (.30)
**Payoff Variability**				
$1.60	.47 (.16)	.33 (.16)	.43 (.26)	.36 (.23)
$4.50	.40 (.14)	.42 (.18)	.47 (.26)	.35 (.24)
$9.60	.42 (.20)	.42 (.18)	.40 (.24)	.43 (.26)

In the second analysis, we collapsed across the levels of *p*_desirable_ and conducted a 2 (time pressure) × 2 (framing) × 3 (payoff variability) mixed-design ANCOVA with Self-Control as a covariate on the proportion of risky choices taken. As previously described, the main effects of time pressure and framing were not significant, and neither was the main effect of payoff variability. The Time Pressure × Payoff Variability interaction was significant, *F*(2, 94) = 4.82, *p* = .01, partial η^2^ = .10 (see Table 5). This interaction was driven by a difference at the lowest ($1.60) level of payoff variability, *t*(47) = 3.10, *p* = .003, *d* = .88, with participants taking the risky option more often in the high time-pressure condition (*M* = .47) than in the low time-pressure condition (*M* = .33). Differences in choice behaviour in the other two levels of payoff variability were not significant. The interaction of Framing × Payoff Variability was significant, *F*(1.76, 80.85) = 4.28, *p* = .017, partial η^2^ = .08 (see Table 5), after correction for non-sphericity using the Greenhouse-Geisser estimate (ε = .88). The interaction was driven by a difference at the medium ($4.50) level of payoff variability, *t*(48) = 2.20, *p* = .032, *d* = .48, with participants taking the risky option more often for gains (*M* = .47) than for losses (*M* = .35).

#### Information search

**Fig 3:**
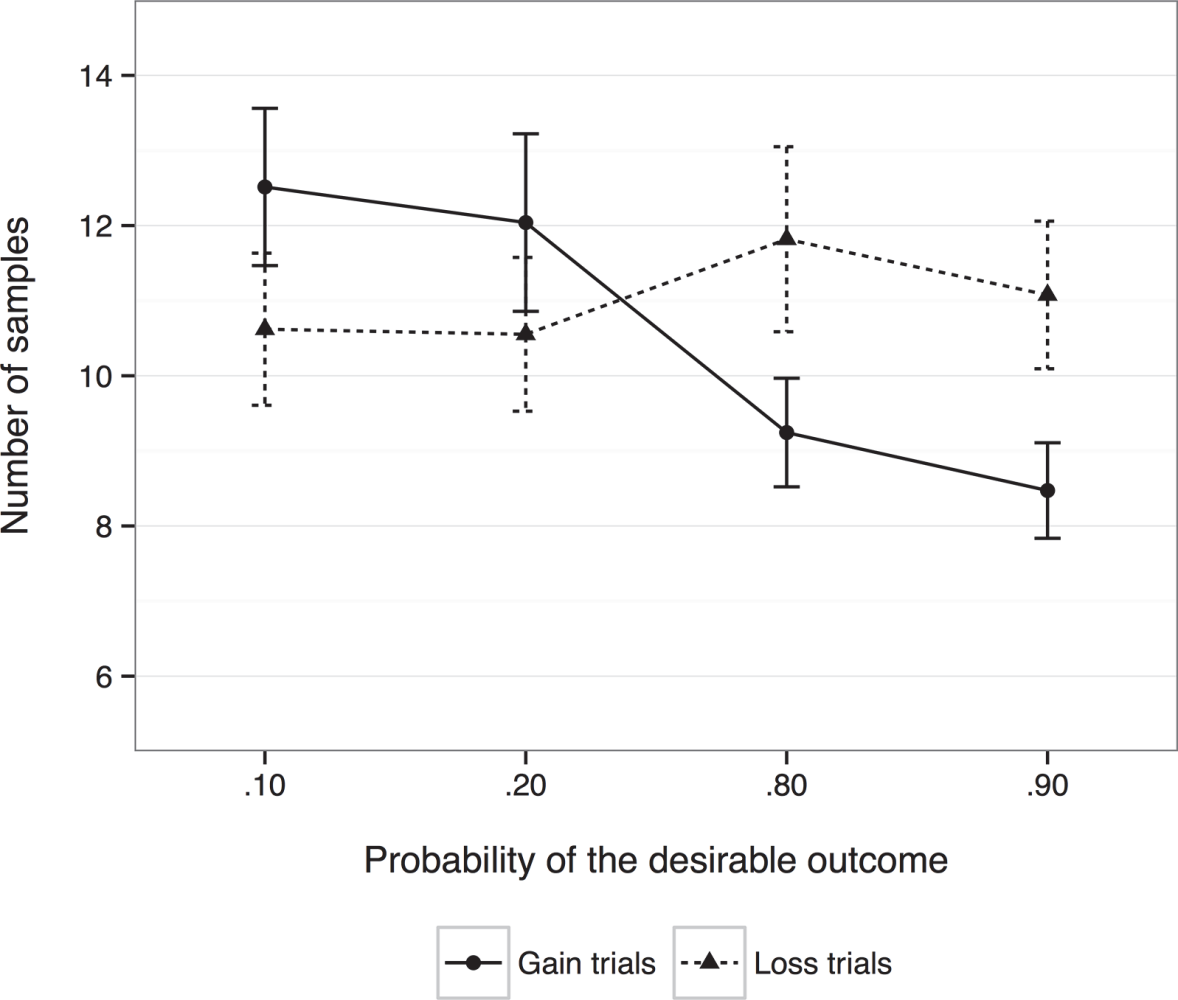
Experiment 2—Number of samples taken for each level of *p*_desirable_ as a function of choice framing (gains vs. losses). Error bars indicate standard errors of the mean.

Information search was operationalized as the number of samples participants drew before making a choice. First, we collapsed across the levels of payoff variability and conducted a 2 (time pressure) × 2 (framing) × 4 (*p*_desirable_) mixed-design ANCOVA with Self-Control as a covariate to investigate degree of information search. The main effect of time pressure was significant, *F*(1, 46) = 7.08, *p* = .01, partial η^2^ = .13, with participants drawing more samples during the low time-pressure condition (*M* = 12.9) than during the high time-pressure condition (*M* = 8.6). The main effects of framing and *p*_desirable_ were not significant and neither were any interactions with time pressure. A significant Framing × *p*_desirable_ interaction was found, *F*(2.07, 95.39) = 8.81, *p* < .001, partial η^2^ = .16, after correction for non-sphericity using the Greenhouse-Geisser estimate (ε = .69). Planned contrasts revealed a significant linear relationship during gain trials, *F*(1, 46) = 21.00, *p* < .001, partial η^2^ = .31, with participants sampling less often as the level of *p*_desirable_ increased. There were no significant relationships for loss trials (Fig 3).

Second, we collapsed across the levels of *p*_desirable_ and conducted a 2 (time pressure) × 2 (framing) × 3 (payoff variability) mixed-design ANCOVA with Self-Control as a covariate. Only the main effect of payoff variability was significant, *F*(2, 92) = 5.12, *p* = .008, partial η^2^ = .10, and planned linear contrasts showed participants sampled more often as the payoff variability increased, *F*(1, 46) = 11.03, *p* = .002, partial η^2^ = .19. No interactions were significant.

Finally, to account for differences in information between time pressure conditions and the effect this may have had on risk-taking, we ran additional ANCOVA on the proportion of risking choices, with sample size as a mean-centered covariate. We found the same pattern of results as before—a difference between time-pressure conditions at the. 90 level only, with greater risk seeking behaviour under high time pressure than under low time pressure. This suggests that the differences in sample size due to time pressure did not influence participants’ weightings of rare events. Along similar lines, Hau et al. found that sample size had little impact on the propensity of participants to select the risky option, with sample sizes of 5, 10, 20, 35, and 50, with almost no difference whatsoever across sample sizes. These points suggest that the observed differences in sample size between time pressure conditions did not affect risk-seeking behaviour in participants.

#### Correlations with self-report measures

We performed bivariate correlations of participants’ self-report measures with their risk-taking, information search, and reaction times. We found that Risk Propensity (RP) and Self-Control (SC) were significantly correlated with information search. Participants who had higher RP scores—more proneness to risk-seeking behaviour—sampled fewer times than participants with lower RP scores, *r* = -.38, *p* = .007. Participants with higher SC scores—more self-control—sampled more often than those with lower SC scores, *r* = .37, *p* = .009. Both of these measures also correlated with reaction times but as reaction time in the sampling paradigm is confounded with information search, these correlations are not reported separately.

#### The description-experience gap

**Fig 4:**
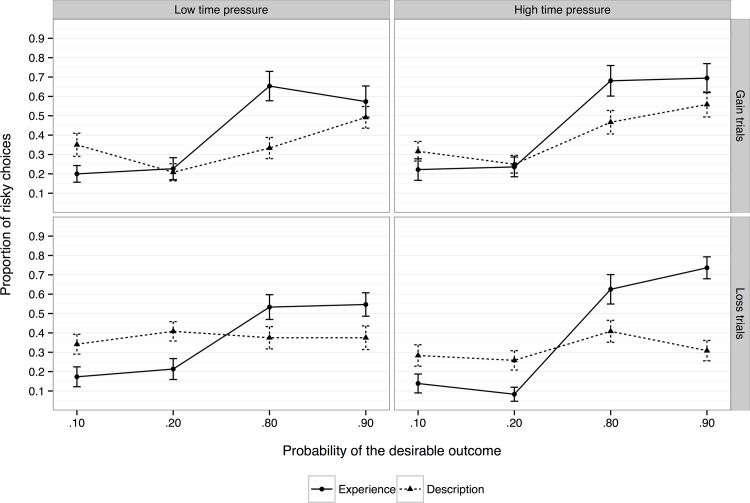
Comparison of choice proportions between conditions (description vs. experience) as a function of time pressure condition and choice framing.

We did not originally set out to investigate the role of time pressure on the description-experience gap. Time pressure was manipulated within subjects in Experiment 1 but between subjects in Experiment 2, making it difficult to compare the time-pressure effect directly across the two experiments. Nevertheless, a qualitative inspection of the time-pressure effects is informative (Fig 4). The upper left panel plots participants’ risk-taking on gain trails in the low time pressure condition for both experiments, and is similar to Hau et al.’s [19] findings. While we did replicate their pattern of results for the experience-based experiment, we did not in the description-based experiment and thus did not find the classic description-experience gap. Risk-taking has been found to decrease as *p*_desirable_ increases and to remain flat at the. 80 and. 90 levels in Hau et al. [19]; however, risk-taking in our study increased from the. 20 level onward. The upper right plots risk-taking in the high time pressure condition. Here we see that under high time pressure description-based choice risk-taking increases and appears more like experience-based choice (i.e., increased time pressure closes the description-experience gap and results in more experience-like behaviour in the description condition). The lower plots compare description and experience in the two time pressure conditions for loss trials. We see roughly the same pattern of results for experience-based choice as for gains, but behaviour in loss trials is essentially flat across levels of *p*_desirable_. Thus there is tentative evidence that time pressure may close the description-experience gap in gain trials while loss trials seem less affected; however, further research is necessary before firm conclusions can be drawn.

### Discussion

In Experiment 2, we manipulated time pressure between subjects in the risky financial choice task. Decisions were made from experience rather than description, which provided us with a new way to compare participant behaviour between time-pressure conditions, namely through the degree of information search engaged in by participants prior to making a final decision. While the time-pressure manipulation was successful at modulating response times, we found no effects of time pressure or choice framing on risk-taking. Similar to Experiment 1, we found evidence of adaptive decision making as participants increased their risk-taking as *p*_desirable_ increased. Specifically, participants displayed greater risk-taking at the. 90 level when under high time pressure than under low time pressure. Additionally, when payoff variability was low, participants made more risky choices under high time pressure than under low time pressure.

Our analysis revealed that time pressure affected information search, with participants sampling less when time pressure was high compared with when it was low. Based on past literature [5,6,20], we had hypothesized that participants would sample more on loss trials than on gain trials in decisions from experience. Although there was no main effect of framing on information search behaviour, we found a significant interaction of framing and *p*_desirable_, revealing that participants searched for less information during gain trials as the probability of receiving the desirable outcome increased. This pattern was similar to the previously observed adaptive sampling [23] although in that study we also found decreased sampling for losses as the probability of receiving the desirable outcome increased, while here we found no significant difference on sampling behaviour for loss trials as *p*_desirable_ increased.

## General Discussion

In this study we sought to investigate the effects of time pressure on risk-taking and information search in the context of financial decisions. Participants were presented with a series of problems requiring a choice between smaller-but-certain and larger-but-risky monetary rewards. Options varied in choice framing (gain vs. loss), the probability of receiving the desirable—gain or non-loss—outcome (.10,. 20,. 80,. 90), and the payoff variability of the risky option ($1.60, $4.50, $9.60). Participants completed the choice problems with or without time constraints. The time constraints were individually calibrated for each participant on the basis of response times during a practice phase. In Experiment 1, choices were presented in a description-based format where probability information was explicitly provided to the participant, and each participant completed the task under high and low time-pressure conditions. In Experiment 2, choices were presented in an experience-based format that required participants to learn the probability of the risky option through a sequential sampling procedure. Time pressure was manipulated between subjects.

In Experiment 1, we found a significant interaction between time pressure and the framing (gain vs. loss) of the choice. For gains, participants tended to select the risky option more often in the high time pressure condition than the low time pressure condition, although this trend failed to reach statistical significance. For losses, participants selected the risky option more often in the low time pressure condition than the high time pressure condition. Contrary to our Framing hypothesis, when acting under a time constraint participants became more risk seeking for gains and less risk seeking for losses, relative to the low time pressure condition.

In line with our Adaptivity hypothesis, we observed a significant modulation of risk-taking according to the probability of the desirable outcome, whereby participants chose the risky option more often as *p*_desirable_ increased. This is in contrast to previous findings in the literature which found that the risky option was selected less often as the probability of the desirable outcome increased [19]. Finally, we found that participants with higher Need for Cognition scores displayed greater risk-seeking behaviour.

In Experiment 2, time pressure, choice framing, and their interaction had no impact on risk-taking, suggesting that time constraints do not significantly alter risk attitudes in the experience-based choice domain. This is especially interesting insofar as time pressure did have a significant effect on pre-decisional information search in line with our Adaptivity hypothesis, with less search in the high time-pressure condition compared with the low time-pressure condition. We found the same pattern of results when controlling for sample size. Thus, restricted sampling due to a time constraint does not significantly impact subsequent decision-making behaviour. Reliance on small samples has previously been observed in the literature [17,19,23] with the relationship between sample size and choice being a weak one [31]. Additionally, we observed two significant correlations: Participants with higher Risk Propensity scores engaged in less information search, whereas higher Self-Control scores correlated with greater information search. This suggests that information seeking in experience-based choice may be partly determined by personality traits. Comparing the two experiments, we found tentative evidence that increased time pressure may close the description-experience gap—at least for choices involving gains—but as our studies were not designed to explicitly investigate it may be an artifact of the within-subjects time pressure manipulation used. Further work is needed to investigate this possibility.

One limitation of our work concerns the degree of time pressure experienced by participants in both experiments. Although time constraints were tailored to each participant, the level used was fairly conservative (75^th^ percentile of response times from practice trials). Furthermore, we did not measure the participants’ subjective experience of time pressure. These two factors may place limitations on the applicability of the current findings to real-life scenarios in which financial decisions are made under time pressure, such as trading on the stock market. In particular, the time pressure experienced by participants in our study may be weaker than that experienced by professional traders. Future work in this area should investigate how the severity of time constraints affects both risk-seeking and pre-decisional information search behaviour across both description- and experienced-based choice paradigms. In addition, it would be interesting to examine the role of expertise in decision making under stress, for example by comparing laypeople and financial-sector professionals with respect to their performance in tasks such as those used in the current paper.

Another limitation of the current study is the fact that both experiments involved mostly-female samples, as is often the case in studies using undergraduate psychology student populations. We had no *a priori* hypotheses regarding gender differences in the effects of time pressure on risky choice [32,33], but we nevertheless conducted exploratory analyses to see whether gender played a role in the current study. These analyses yielded no statistically significant effects, nor any statistical trends; however, we acknowledge that the statistical power to detect such trends was limited. The inclusion of gender-balanced samples is a desirable goal for future studies in this domain.

The current study is the first to examine the impact of time pressure on both description- and experience-based risky financial choices. We found that in description-based choice the introduction of a time constraint decreased risk-taking in the face of losses. For experience-based choice, while time constraints did decrease the degree of pre-decisional information search engaged in by participants, no effect was seen on subsequent choice. Additionally, an informal exploration of the description-experience gap across the two experiments suggested that time pressure may have modulated this gap, with choice behaviour in the description-based format appearing more like behaviour in the experience-based format. Overall, the current results suggest that stable choice preferences—ones that do not easily vary as a result of environmental factors such as time pressure—may best be elicited through the use of experience-based choice paradigms.
